# Amelioration of Dextran Sodium Sulfate-Induced Colitis in Mice by *Rhodobacter sphaeroides* Extract

**DOI:** 10.3390/molecules171113622

**Published:** 2012-11-16

**Authors:** Wen-Sheng Liu, Man-Chin Chen, Kuo-Hsun Chiu, Zhi-Hong Wen, Che-Hsin Lee

**Affiliations:** 1 Department of Marine Biotechnology and Resources, National Sun Yat-sen University, Kaohsiung 804, Taiwan; 2 Asia-Pacific Biotech Developing, Inc. Kaohsiung 806, Taiwan; 3 Graduate Institute of Basic Medical Science, School of Medicine, China Medical University, Taichung 404, Taiwan; 4 Department and Graduate Institute of Aquaculture, National Kaohsiung Marine University, Kaohsiung 404, Taiwan; 5 Department of Microbiology, School of Medicine, China Medical University, Taichung 404, Taiwan

**Keywords:** *Rhodobacter sphaeroides*, Lycogen™; dextran sodium sulfate, colitis, inflammatory

## Abstract

Bacteria can produce some compounds in response to their environment. These compounds are widely used in cosmetic and pharmaceutical applications. Some probiotics have immunomodulatory activities and modulate the symptoms of several diseases. Autoimmune diseases represent a complex group of conditions that are thought to be mediated through the development of autoreactive immunoresponses. Inflammatory bowel disease (IBD) is common autoimmune disease that affects many individuals worldwide. Previously, we found that the extracts of *Rhodobacter sphaeroides* (Lycogen) inhibited nitric oxide production and inducible nitric-oxide synthase expression in activated macrophages. In this study, the effect of Lycogen™, a potent anti-inflammatory agent, was evaluated in mice with dextran sodium sulfate (DSS)-induced colitis. Oral administration of Lycogen™ reduced the expressions of proinflammatory cytokines (tumor necrosis factor-α and interleukin-1β) in female BABL/c mice. In addition, the increased number of bacterial flora in the colon induced by DSS was amelirated by Lycogen™. The histological score of intestinal inflammation in 5% DSS-treated mice after oral administration of Lycogen™ was lower than that of control mice. Meanwhile, Lycogen™ dramatically prolonged the survival of mice with severe colitis. These findings identified that Lycogen™ is an anti-inflammatory agent with the capacity to ameliorate DSS-induced colitis.

## 1. Introduction

Autoimmune diseases represent a complex group of conditions that are thought to be mediated through the development of autoreactive T cells or antibodies [1]. Clinically, the etiology of autoimmune diseases is often complex; the majority are chronic conditions lasting a lifetime. Treatment of these diseases usually relies on non-specific inhibition of inflammatory immune responses [2]. Anti-inflammatory agents derived from nature products may be an alternative, promising modality for efficient, long-term, and safe treatments. Inflammatory bowel disease (IBD) is common autoimmune diseases that affect many individuals worldwide. IBD involves a complex interplay between certain genetic, environmental, and immunological factors. Proinflammatory cytokines and angiogenic factors play a key role in the pathogenesis of IBD [3]. The abnormal reactivity to luminal or mucosal antigens in IBD may be the result of an imbalance between proinflammatory and regulatory CD4^+^ T lymphocytes and Th1-associated inflammatory cytokines [4]. Tissue and serum levels of proinflammatory cytokines have been reported to be higher in active IBD [5].

Some nutritional and natural components have been studied for their effectiveness in treating IBD by controlling production of proinflammatory cytokines [4,5,6,7,8,9]. Furthermore, microorganisms such as fungi and bacteria produce many metabolites that have been developed into drugs. Bacteriopurpurinimides were derived from the phototrophic bacteria, *Rhodobacter sphaeroides* [10]. Carotenoids are naturally occurring compounds also found in *R. sphaeroides* [11]. Studies found that carotenoids having anti-oxidant activity are able to inhibit various types of cancer [12]. Therapeutic strategies utilizing nature products may enhance mucosal immune balance and increase therapeutic response. In this study, we identified a novel strategy by exploiting the extract of *R. sphaeroides* (Lycogen™), aiming at treating murine experimental colitis using its anti-inflammatory effects.

## 2. Results

### 2.1. Lycogen™ Reduced the Expression of Proinflammatory Cytokines in Mice with DSS-Induced Colitis

**Figure 1:**
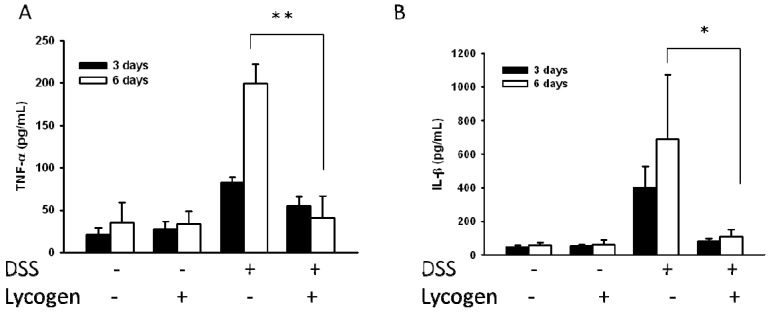
Effects of Lycogen™ on cytokine induction in DSS-induced colitis. The mice with colitis were orally administered with Lycogen™ (1 mg/kg) for six consecutive days after DSS induction. (**A**) TNF-α and (**B**) IL-1 β levels in the sera were measured by ELISA at day 6 (mean ± SD, n = 4). Significance of differences between groups for continuous variables was assessed using the Student t-test. * *p* < 0.05; ** *p* < 0.01. Each experiment was repeated three times with similar results.

We evaluated the effect of Lycogen™ on the productions of proinflammatory cytokines linked to DSS-induced colitis. As shown in Figure 1, DSS induced protein expression of proinflammatory cytokines, such as IL-1β and TNF-α, in sera, whereas Lycogen™ suppressed the host protein expressions of proinflammatory cytokines. These results pointed out that oral administration of Lycogen™ inhibited the production of proinflammatory cytokine in the DSS-induced colitis model.

### 2.2. Lycogen™ Improved DSS-Induced Body Weight Loss and Survival

**Figure 2:**
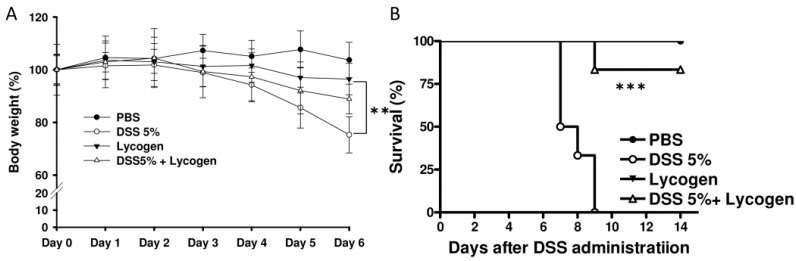
Effect of Lycogen™ on DSS-induced colitis mice. (**A**) The mice with colitis were orally administered with Lycogen™ (1 mg/kg) for six consecutive days after DSS induction and the body weights of mice were determined (means ± SD, n = 6). *p* < 0.01 for colitis mice treated with Lycogen™ *versus* colitis mice treated with PBS (**B**) Kaplan-Meier survival curves on day 14 are shown. (mean ± SD, n = 6) Significance of differences between groups for continuous variables was assessed using the Student t-test. The mice survival analysis was performed using the Kaplan-Meier survival curve and log-rank test. ** *p* < 0.01; *** *p* < 0.001.

We further monitored symptomatic colitis parameters including body weight and survival caused by colitis 6 days after starting 5% DSS administration (Figure 2). Treatment of mice with Lycogen™ significantly attenuated the weight loss induced by DSS (Figure 2A). Oral Lycogen™ did not influence the body weight and survival of mice, suggesting that Lycogen™ was safe for mice. Furthermore, Lycogen™ dramatically prolonged the survival of mice with severe colitis (Figure 2B).

### 2.3. Lycogen™ Suppressed DSS-Induced Colitis in Mice

**Figure 3:**
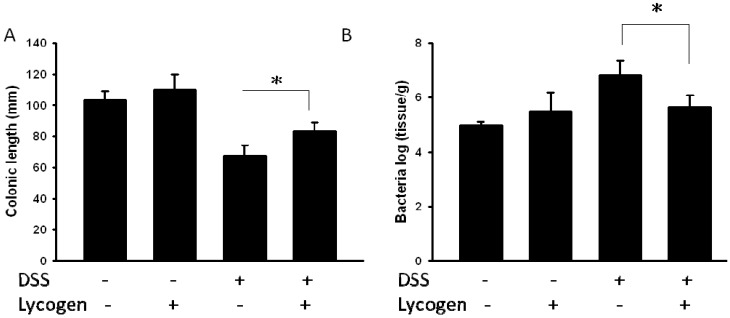
Lycogen™ inhibited DSS-induced colonic shorting and bacterial flora. (**A**) The mice with colitis were orally administered with Lycogen™ (1 mg/kg) for six consecutive days after DSS induction and the colon lengths were determined at day 6 (means ± SD, n = 4). (**B**) The mice with colitis were orally administered with Lycogen™ (1 mg/kg) for six consecutive days after DSS induction and the amount of bacteria in colon was determined. Significance of differences between groups for continuous variables was assessed using the Student t-test. * *p* < 0.05.

Colon lengths were measured due to the observation that a short colon can be used as a morphological indicator of colon inflammation in DSS-treated mice [13]. As shown in Figure 3A, the colon length of mice treated with DSS was significantly shortened compared with the control mice. The length of colon in the oral administration of Lycogen™ group was longer than that in the DSS-treated group. Bacterial load and damage scores were significantly correlated with acute colitis. The number of bacteria in mice with colitis was significantly higher than in control mice. However, there were significantly more bacteria in colitis mice treated with PBS than that in colitis mice treated with Lycogen™ (Figure 3B).

**Figure 4:**
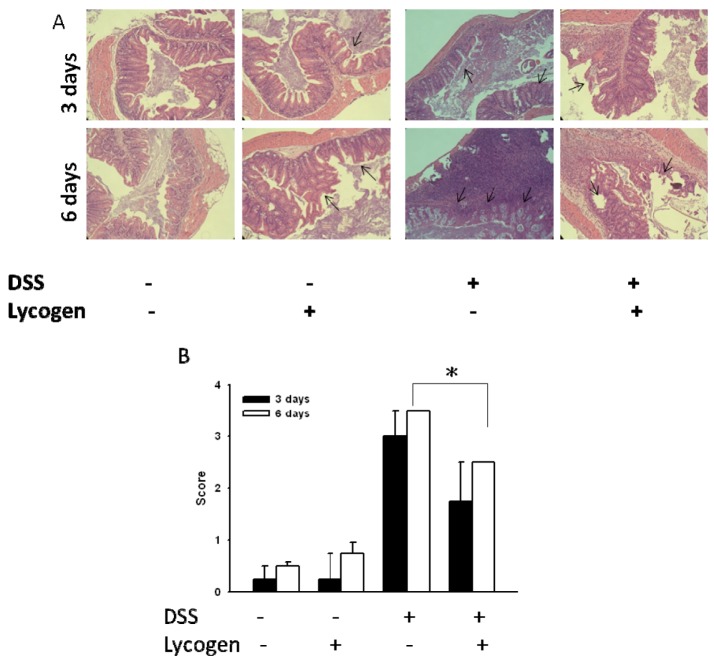
Therapeutic effect of Lycogen™ in mice with DSS-induced colitis. Mice were orally administered Lycogen™ (1 mg/kg) for six consecutive days after DSS induction. (**A**) Microscopic features of the colon at day 3 and day 6. Arrows indicate the damaged sites. (**B**) The microscopic score of DSS-induced colitis (means ± SD, n = 4). Significance of differences between groups for continuous variables was assessed using the Student t-test. * *p* < 0.05.

Next, we evaluated the effect of Lycogen™ on DSS-induced colitis. As shown in Figure 4, Lycogen™ improved the histological features of DSS-induced colitis. Histological examination of colons from DSS-induced mice showed mucosal thickening and an increase in infiltrating lymphocytes (Figure 4A). Lycogen™ significantly reduced the histological severity compared with the control groups. These findings suggested the therapeutic potential of Lycogen™ in DSS-induced colitis.

## 3. Discussion

Probiotics are beneficial for maintaining intestinal homeostasis [14]. The current study demonstrated that a bacteria-derived molecule could improve the epithelial cell injury caused by intestinal inflammation [15]. Experimental colitis produced by DSS is thought to share many important characteristics with forms of human inflammatory bowel disease. In this study, we used the extract of *R. sphaeroides* (Lycogen™) to show its bioactivity in DSS-induced colitis. Our results showed that Lycogen™ was capable of improving colonic damage, suggesting that it could be a potent therapeutic agent for the treatment of IBD.

Mice fed diets containing Lycogen™ (1 mg/kg) for 6 days showed no effect on body weight, proinflammatory cytokine productions, and no microscopic changes in colonic tissue. Moreover, no adverse side effects were observed when the diet contained Lycogen™ (Figure 2B). NO overreaction was observed in patients with IBD. Lycogen™ inhibited NO production and inducible nitric-oxide synthase (iNOS) expression in activated macrophage [16]. These findings implied that the anti-oxidative potential of Lycogen™ have the activity to inhibit the DSS-induced colitis. Inflammatory cytokines play important role in the pathogenesis of IBD. High levels of the pro-inflammatory cytokines, IL-1β and TNF-α, are present in the gut mucosa of patients suffering from IBD. Both TNF-α and IL-1β have been implicated in stimulating the production of IL-8 in the pathogenesis of IBD [17]. The levels of TNF-α and IL-1β were significantly raised in DSS-induced colitis [4]. Oral administration of Lycogen™ prevented the rise in both TNF-α and IL-1β. Short-chain fatty acids (SCFA) are produced by fermentation of water-soluble fiber by anaerobic bacteria. Butyrate is a major SCFA produced by microbial fermentation of dietary fiber in the gastrointestinal tract. SCFA significantly inhibited the pro-inflammatory cytokines expression [18]. Butyrate decreases pro-inflammatory cytokine expression via inhibition of NF-κB activation [19]. TNF-α and IL-1β involved in the development of DSS-induced colitis in mice. The levels of TNF-α and IL-1β in sera might explain the therapeutic effect of Lycogen™.

Lycogen™ contains ζ-carotene, neurosporene, spheroidenone and methoxyneurosporene accordinh to nuclear magnetic resonance spectroscopy analysis. ζ-Carotene is the precursor of neurosporene, which in turn is the precursor of lycopene [20]. Lycopene, as an anti-inflammatory agent, prevents the production of inflammatory cytokines [21]. Meanwhile, neurosporene itself has the ability to protect irradiation with UV-B [22]. Further work is warranted to elucidate the active ingredient(s) in Lycogen™. These findings point out that the anti-oxidative and anti-inflammatory potential of Lycogen™ might contribute to its therapeutic effect on DSS-induced colitis.

## 4. Experimental

### 4.1. Reagents and Mice

*R. sphaeroides* (WL-APD911) was isolated from mutants using chemical mutagenesis [Bioresource Collection and Research (BCRC), Hsinchu, Taiwan]. The *R. sphaeroides* was cultured in broth. After harvesting, the bacterial broth was centrifuged and washed with ethanol. The bacterial residue is extracted with acetone and then centrifuged by 7,500 rpm for 5 min. The supernatant is filtered through filter paper and a 0.2 μm filter into a round-bottomed flask. The color of the final supernatant is dark red. Acetone is removed completely in oven at 55 °C. The *R. sphaeroides* extract was named Lycogen™ [16]. Lycogen™ was dissolved in PBS. Female BALB/c mice were obtained from the Laboratory Animal Center of the National Cheng Kung University. The experimental protocol adhered to the rules of the Animal Protection Act of Taiwan. The experimental protocol has been approved by the Laboratory Animal Care and Use Committee. All mice were kept under standard conditions of temperature and light, and were fed with standard laboratory chow and water. Dextran sodium sulfate (DSS) with molecular weight of 36,000~50,000 was purchased from MP Biomedicals (Solon, OH, USA).

### 4.2. Establishment of Experimental Colitis Model

Female BABL/c mice at 6–8 weeks of age were given 5% DSS in their drinking water for six days as previously described [23]. To study the therapeutic effect of Lycogen™, this substance (1 mg/kg/day) was orally administered for six consecutive days by gavage. Control mice were treated with PBS. Body weight loss is calculated as the percent difference between the original body weight and the actual body weight daily.

### 4.3. Bacteria Culture

Mice were induced colitis and colons were collected at day 6. Samples were homogenised and serially diluted in PBS. After serial dilution of the colonic suspensions with appropriate buffers, the diluent were spread onto LB plates. The plates were cultured at 37 °C. Bacterial numbers were expressed as log10 counts of viable bacteria/g wet weight of colonic contents.

### 4.4. Assessment of Cytokines

To determine the expression of tumor necrosis factor-α and interleukin-1β, mice were induced with 5% DSS in their drinking water for six days. Then, the groups of mice were treated with Lycogen™ (1 mg/kg) by oral administration daily for six consecutive days by gavage. To detect cytokine expressions, the sera were collected at day 6. Levels of cytokines in the sera were determined by an enzyme-linked immunosorbent assay (ELISA, R&D, Minneapolis, MN, USA).

### 4.5. Assessment of Clinical Colitis and Histopathology Score

At the indicated time points after therapy, mice with experimental colitis were sacrificed, and explanted colons were formalin-fixed, paraffin-embedded, and sectioned. Sections were subjected to H & E staining. Histological scoring is performed on operator-blinded sections using the standardized histological point system described in [24], which is used routinely for histological scoring of IBD severity. A score of 0 reflects normal epithelium, without blunting, normal crypt appearance, low monocyte infiltration, and low or absent neutrophil infiltration. Three serial sections of five to six different sites of the colon (accounting for up to 18 sections per mouse) are examined at 200× magnification; the most affected part is scored, ulceration being considered the worst lesion. A score of 1 indicates loss of single epithelial cells, mild blunting of the epithelium, single inflammatory cell infiltration of crypts, slight monocyte, and neutrophil infiltrate; a score of 2 signifies loss of multiple epithelial cells (in patches), obvious flattening of the epithelia, cryptitis, and a moderate increase in monocytes and neutrophils; a score of 3 indicates frank epithelial ulceration with crypt abscesses and a marked increase in monocyte/neutrophils.

### 4.6. Statistical Analysis

All data were expressed as mean ± standard deviation (SD). The unpaired, two-tailed Student’s t test was used to determine differences between groups. The mice survival analysis was performed using the Kaplan-Meier survival curve and log-rank test. Any *p* value less than 0.05 is considered statistically significant.

## 5. Conclusions

In conclusion, our work has identified Lycogen™ as an anti-inflammatory agent with the capacity to ameliorate DSS-induced colitis. However, further work is warranted to elucidate the underlying mechanism of the therapeutic effects of Lycogen™ therapy in the DSS-induced colitis model.
